# Mechanical thrombectomy in acute basilar artery stroke: a systematic review and Meta-analysis of randomized controlled trials

**DOI:** 10.1186/s12883-022-02953-2

**Published:** 2022-11-09

**Authors:** Abid Malik, Brian Drumm, Lucio D’Anna, Isabelle Brooks, Benjamin Low, Oishik Raha, Khawar Shabbir, Orsolya Vittay, Joseph Kwan, Zoe Brown, Omid Halse, Sohaa Jamil, Dheeraj Kalladka, Marius Venter, Harri Jenkins, Neil Rane, Abhinav Singh, Maneesh Patel, Charles Hall, Gavin Fatania, Dylan Roi, Kyriakos Lobotesis, Soma Banerjee

**Affiliations:** Stroke Centre, Department of Stroke and Neuroscience, Charing Cross Hospital, Imperial College London NHS Healthcare Trust, Fulham Palace Road, London, W6 8RF UK

**Keywords:** Basilar, Stroke, Meta-analysis, Mechanical thrombectomy, Randomized controlled trials

## Abstract

**Background:**

The evidence for mechanical thrombectomy in acute basilar artery occlusion has until now remained inconclusive with basilar artery strokes associated with high rates of death and disability. This systematic review and meta-analysis will summarize the available evidence for the effectiveness of mechanical thrombectomy in acute basilar artery occlusion compared to best medical therapy.

**Methods:**

We conducted a systematic review and meta-analysis of randomized controlled trials using Embase, Medline and the Cochrane Central Register of Controlled Trials (CENTRAL). We calculated risk ratios (RRs) and 95% confidence intervals (CIs) to summarize the effect estimates for each outcome.

**Results:**

We performed a random effects (Mantel-Haenszel) meta-analysis of the four included randomized controlled trials comprising a total of 988 participants. We found a statistically significant improvement in the rates of those with a good functional outcome (mRS 0–3, RR 1.54, 1.16–2.06, *p* = 0.003) and functional independence (mRS 0–2, RR 1.69, 1.05–2.71, *p* = 0.03) in those who were treated with thrombectomy when compared to best medical therapy alone. Thrombectomy was associated with a higher level of sICH (RR 7.12, 2.16–23.54, *p* = 0.001) but this was not reflected in a higher mortality rate, conversely the mortality rate was significantly lower in the intervention group (RR 0.76, 0.65–0.89, *p* = 0.0004).

**Conclusions:**

Our meta-analysis of the recently presented randomized controlled studies is the first to confirm the disability and mortality benefit of mechanical thrombectomy in basilar artery stroke.

**Supplementary Information:**

The online version contains supplementary material available at 10.1186/s12883-022-02953-2.

## Background

The basilar artery is the primary blood supply to the posterior circulation supplying the brain stem, occipital lobes, thalami and cerebellum. Acute basilar artery occlusion (BAO) is a rare but devastating cause of stroke with a mortality rate of up to 45% [1]. Previous randomized controlled trials have formed the basis for the use of mechanical thrombectomy (MT) in the anterior circulation and have revolutionized acute stroke treatment [2, 3], however only one of these trials, THRACE (Mechanical thrombectomy after intravenous alteplase versus alteplase alone after stroke) included a small number of patients with BAO (4 patients in total) [4].

Two recent randomized controlled trials, BEST (Basilar Artery Occlusion Endovascular Intervention Versus Standard Medical Treatment) [5] and BASICS (Basilar Artery International Cooperation Study) [6] failed to show a benefit in terms of functional disability and mortality at 90 days, however these trials were plagued with slow recruitment (2011–2019 in BASICs and 2015–2017 in BEST), large numbers of patients treated outside of the trial protocol (29% in BASICS and 55% in BEST) and suffered from high crossover rates (22% in BEST). The lack of a positive outcome was disappointing and unexpected given the high rates of recanalization and the poor prognosis associated with basilar artery occlusion.

The research landscape has now changed significantly following the presentation of the first two positive randomized controlled trials, ATTENTION (Endovascular treatment for acute basilar artery occlusion) [7, 8] and BAOCHE (Basilar Artery Occlusion Chinese Endovascular trial) [9, 10] at the European Stroke Conference (2022) [11]. We present a meta-analysis of the combined results from all four randomized controlled trials including the presented data in order to better understand the role of MT in BAO.

## Methods

### Protocol and guidance

The meta-analysis conducted adheres to the PRISMA statement (Preferred Reporting Items for Systematic Reviews and Meta-Analyses) [12]. We included randomized controlled trials with a parallel design that randomized participants to BMT and MT versus BMT alone. See Table 1 for the PICO framework.

**Table 1:** PICO table, MT for Basilar artery occlusion

**Population**	Acute ischemic stroke due to basilar artery occlusion.
**Intervention**	Mechanical thrombectomy and best medical therapy.
**Comparison**	Best medical therapy only.
**Outcome**	Good neurological outcome defined as a modified Rankin Scale (mRS) of ≤3 at day 90. Secondary outcomes included mortality, symptomatic intracranial hemorrhage (sICH) and functional independence (mRS ≤2).

### Literature search and data extraction

We conducted a systematic review using Embase, Medline and the Cochrane Central Register of Controlled Trials (CENTRAL) from inception to July 2022. Additional file 1 contains the predefined search strategy.

We also searched the following trial registers, the US National Institutes of Health Ongoing Trials Register: ClinicalTrials.gov [13], the World Health Organization International Clinical Trials Registry Platform [14] and reviewed the reference list of key papers. In an effort to identify further published, unpublished, and ongoing trials, we conducted a search of various gray literature sources, dissertation and theses databases, and databases of conference abstracts. No time limits or language restrictions were used.

Eligibility of the studies for the quantitative analysis was rated by four independent readers (AM, BD, BL, and IB). We collated multiple reports of the same study so that each study, not each reference, was the unit of interest in the review. Randomized, single blinded studies were included into the quantitative synopsis if the study followed patients up for at least 90 days using the modified Rankin Scale (mRS) [15] and reported on adverse events and mortality.

Patients must have been included within 24 hours of stroke onset and have a clinical syndrome in keeping with a posterior circulation stroke. We defined BMT as thrombolysis, antiplatelet or antithrombotic treatment. Data inconsistencies in the papers selected for inclusion were discussed and resolved by mutual consensus.

### Risk of bias

We performed a risk of bias assessment for each of the included trials. We assessed the following domains and graded each low, unclear or high.

i. Random sequence generation
ii. Allocation concealment
iii. Blinding of participants and personnel
iv. Blinding of outcome assessment
v. Incomplete outcome data
vi. Selective outcome reporting
vii. Other bias

### Statistical analysis

We used the statistical program Review Manager 5.4 to analyze the results. For the dichotomous outcomes we combined the pooled individual study RRs and 95% confidence intervals (CIs) for each outcome using a random-effects Mantel-Haenszel model given the potential for methodological heterogeneity (see Discussion). In included studies we treated the participant as the unit of analysis. We used both a visual inspection of CIs and the I^2^ statistic to calculate the degree of statistical heterogeneity; where we took an I^2^ value of 50% as an indicator of moderate heterogeneity, and an I^2^ of ≥75% as an indicator of substantial heterogeneity.

## Results of the search

**Fig. 1:**
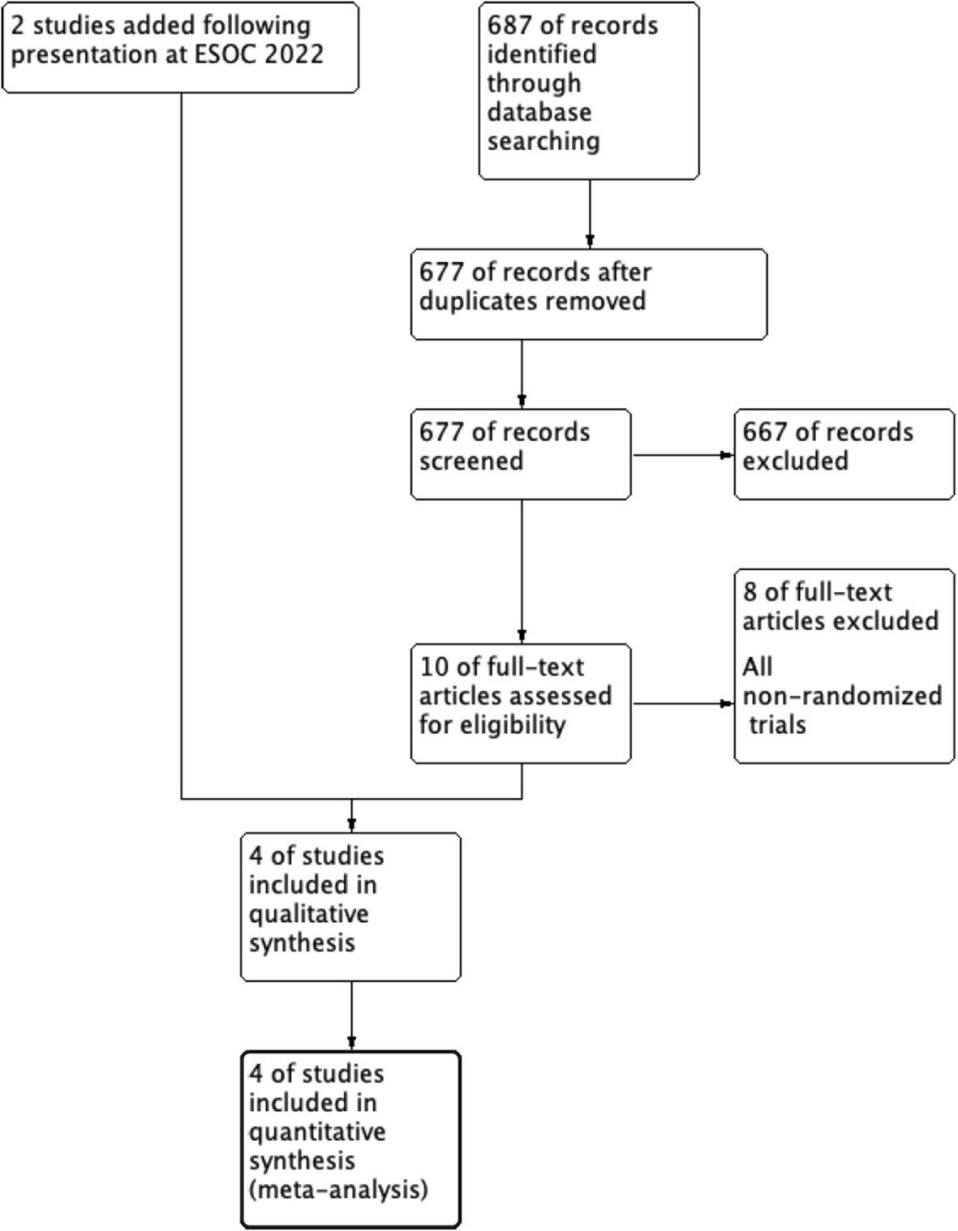
Flow chart according to the Preferred Reporting Items for Systematic Reviews and Meta-Analyses (PRISMA) recommendations

Our database searches retrieved a total of 687 articles, following the review process we identified four randomized controlled trials (see Fig. 1). Two from the initial search strategy and two following the presentation of two randomized controlled trials (ATTENTION [7] and BAOCHE [9]) at the European Stroke Conference [11]. Although the full texts were not available for these articles, we found sufficient information from the presented data in order to conduct a meta-analysis.

### Included studies

Four randomized, controlled, single blinded studies were included in the quantitative analysis [5, 6, 8, 10]. The trials included participants aged greater than 18 years who had a clinical posterior circulation stroke syndrome with confirmed basilar occlusion on CT angiogram or MR angiogram imaging. The trials included participants thrombolysed if presenting with no contraindications. We defined sICH as per the individual trials’ primary analysis (see Table 2).

**Table 2:** Definition of sICH in the included trials

Trial	Definition of sICH
**BASICS**	Heidelberg Bleeding Classification - haemorrhagic transformation of infarcted brain tissue, intracerebral haemorrhage both within and outside infarcted brain tissue, intracerebral haemorrhage outside the infarcted brain tissue, or intracranial–extracerebral haemorrhage and an increase of 4 points or more in the NIHSS score or an increase of 2 points or more in 1 of the 11 NIHSS subcategories.
**BEST**	SITS-MOST criteria - evidence of intracranial haemorrhage on imaging and an increase of 4 or more points on the NIHSS within 24 h after randomisation.
**ATTENTION**	SITS-MOST criteria - evidence of intracranial haemorrhage on imaging and an increase of 4 or more points on the NIHSS.
**BAOCHE**	SITS-MOST criteria - evidence of intracranial haemorrhage on imaging and an increase of 4 or more points on the NIHSS.

### Characteristics of included trials

All four randomized controlled trials have similar study designs (randomized, prospective, open-label trials with blinded outcome assessment) with a primary outcome of a modified Rankin Scale of 0–3 at 90 days (See Table 3: Key characteristics).

**Table 3:** Key characteristics

	BASICS	BEST	ATTENTION	BAOCHE
**Date**	2011–2019	2015–2017	2021–2022	2016–2022
**Symptom onset to inclusion (hours)**	0–6	0–8	0–12	6–24
**Number screened**	424	288	507	Data not available
**Number of participants**	300	131	340	217
**Crossover (percentage)**	3/154 (1.9) to BMT 7/146 (4.7) to MT	3/66 (4.5) to BMT 14/65 (21.5) to MT	3/226 (1.3) to BMT 3/114 (2.6) to MT	1/110 (0.9) to BMT 4/107 (3.7) to MT
**Median NIHSS at presentation (IQR)**	Intervention: 21.9 Control: 22.1 (IQR not available)	Intervention: 32 (18–38) Control: 26 (13–37)	Intervention: 24 (15–35) Control: 24 (14–35)	Intervention: 20 (14.5–29) Control: 19 (12–30)
**Intravenous thrombolysis (%)**	Intervention: 121/154 (78.6) Control: 116/146 (79.5)	Intervention: 18/66 (27) Control: 21/65 (32)	Intervention: 69/226 (30.5) Control: 39/114 (34.2)	Intervention: 15/110 (13.6) Control: 23/107 (21.5)
**Blinding**	Open-label, blinded outcome assessment	Open-label, blinded outcome assessment	Open-label, blinded outcome assessment	Open-label, blinded outcome assessment
**mRS (≤3)** **(percentage)**	Intervention: 68/154 (44.1) Control: 55/146 (37.6)	Intervention: 28/66 (42.4) Control: 21/65 (32.3)	Intervention: 104/226 (46) Control: 26/114 (22.8)	Intervention: 51/110 (46.3) Control: 26/107 (24.2)
**Mortality at day 90 (percentage)**	Intervention: 59 (38.3) Control: 63 (43.2)	Intervention: 22 (33.3) Control: 25 (38.4)	Intervention: 83 (36.7) Control: 63 (55.2)	Intervention: 34 (30.9) Control: 45 (42.1)
**sICH** **(percentage)**	Intervention: 6 (4.5) Control: 1 (0.7)	Intervention: 5 (8) Control: 0	Intervention: 12 (5) Control: 0	Intervention: 6 (8.8) Control: 1 (2.3)
**Follow up:**	24 hrs, 90 days	24 hrs, 90 days	24 hrs, 90 days	24 hrs, 90 days, 6 months, 1 year

### BASICS (basilar artery international cooperation study)

BASICS took place over eight years with 300 participants enrolled and a low crossover rate of 3.33%. The slow recruitment rate of BASICS does call into question the presence of bias in the enrolled population. Trial centers involved in BASICS had high rates of treatment of eligible patients outside the trial (29.2%) of which a high percentage of patients went on to receive endovascular therapy (79%). Furthermore, over the course of the study, there were amendments to the methodology to facilitate recruitment. This led to the inclusion of more mild strokes (NIHSS < 10) which could lead to an undervaluing of the effect of endovascular therapy.

There was no significant difference between the primary outcome measure in the endovascular therapy arm (44.2%) vs best medical therapy (37.7%). Mortality rates were not significantly different (38.3% with intervention and 43.2% with best medical therapy). However, symptomatic ICH rates were higher as expected with endovascular therapy. Whilst not powered, the subgroup analysis of mild strokes does suggest that patients with mild strokes did better with best medical therapy (a high rate of use of intravenous thrombolysis in BASICS, near 80% in both arms) whereas those with larger strokes (NIHSS > 10) did better with endovascular therapy.

### BEST (basilar artery occlusion endovascular intervention vs standard medical treatment)

BEST was a multi-center Chinese trial (28 centers) which took place over 3 years and intended to recruit 288 participants. However, the trial was terminated early with only 131 patients enrolled due to a high crossover rate of 13% (much higher than the other 3 randomized controlled trials discussed in this paper, which all had < 5% crossover rate). When looking at the baseline demographics of the patients recruited, the patients of BEST had a noticeably higher admission NIHSS (32 in intervention and 26 in control) vs other trials which tended to a median NIHSS of 19–22.

With the intention to treat analysis, there was no statistically significant difference between the trial arms. However, a second look accounting for the effect of crossovers (majority of crossovers in this trial were patients who were randomized to best medical therapy alone and went on to have endovascular therapy) showed that more patients achieved the primary outcome with endovascular therapy compared to best medical therapy alone in both per-protocol (44% vs 25%) and as treated populations (47% vs 24%). Unlike ATTENTION and BAOCHE, mortality figures were not different between trial arms despite a higher rate of symptomatic ICH in patients who received endovascular therapy.

### ATTENTION (endovascular treatment of acute basilar artery occlusion)

In comparison to BEST and BASICS, ATTENTION randomized patients with an extended window of 0–12 hours. In contrast to the other studies discussed in this paper, ATTENTION had a randomization rate of 2:1 for intervention to control arms and the trial took place across 36 high volume Chinese thrombectomy centers. One third of patients in both arms received intravenous thrombolysis.

The most statistically significant finding was that 46% of the endovascular therapy group achieved the primary outcome of mRS 0–3 at 90 days compared to only 22.8% in the best medical therapy arm (with an adjusted risk ratio of 2.1, *p* < 0.001). ATTENTION was also positive in its secondary outcomes with patients who received endovascular therapy faring better in terms of overall disability (odds radio 2.8 (1.8–4.4 CI) and more independent functional outcomes (33.2% compared to 10.5% in best medical therapy (adjusted risk ratio 3.2, 1.8–5.4). Similar to BAOCHE, there were more symptomatic intracranial hemorrhagic events. Despite this, mortality was significantly reduced following endovascular therapy (36.7% vs 55.3% with best medical therapy).

### BAOCHE (basilar artery occlusion Chinese endovascular trial)

BAOCHE is the first randomized controlled trial which investigated the outcomes in late presenters with BAO. Patients were only randomized within the 6–24-hour window from symptom onset where they were ineligible for intravenous thrombolysis or had not achieved recanalization after IVT.

Although the trial aimed to recruit 318 patients, the trial was terminated at the planned interim analysis after 217 patients were enrolled due to the statistically significant difference in primary outcome. 46.4% of patients who received endovascular therapy compared to 24.3% of patients who received best medical therapy along achieved the primary outcome with an adjusted odds ratio of 2.92 (1.56–5.47, *p* = 0.001). In terms of safety, whilst the intervention arm had a higher rate of symptomatic intracranial hemorrhages, the overall 90-day mortality was less in the intervention arm (30.9% compared to 42.1% in the control arm).

## Results

**Fig. 2:**
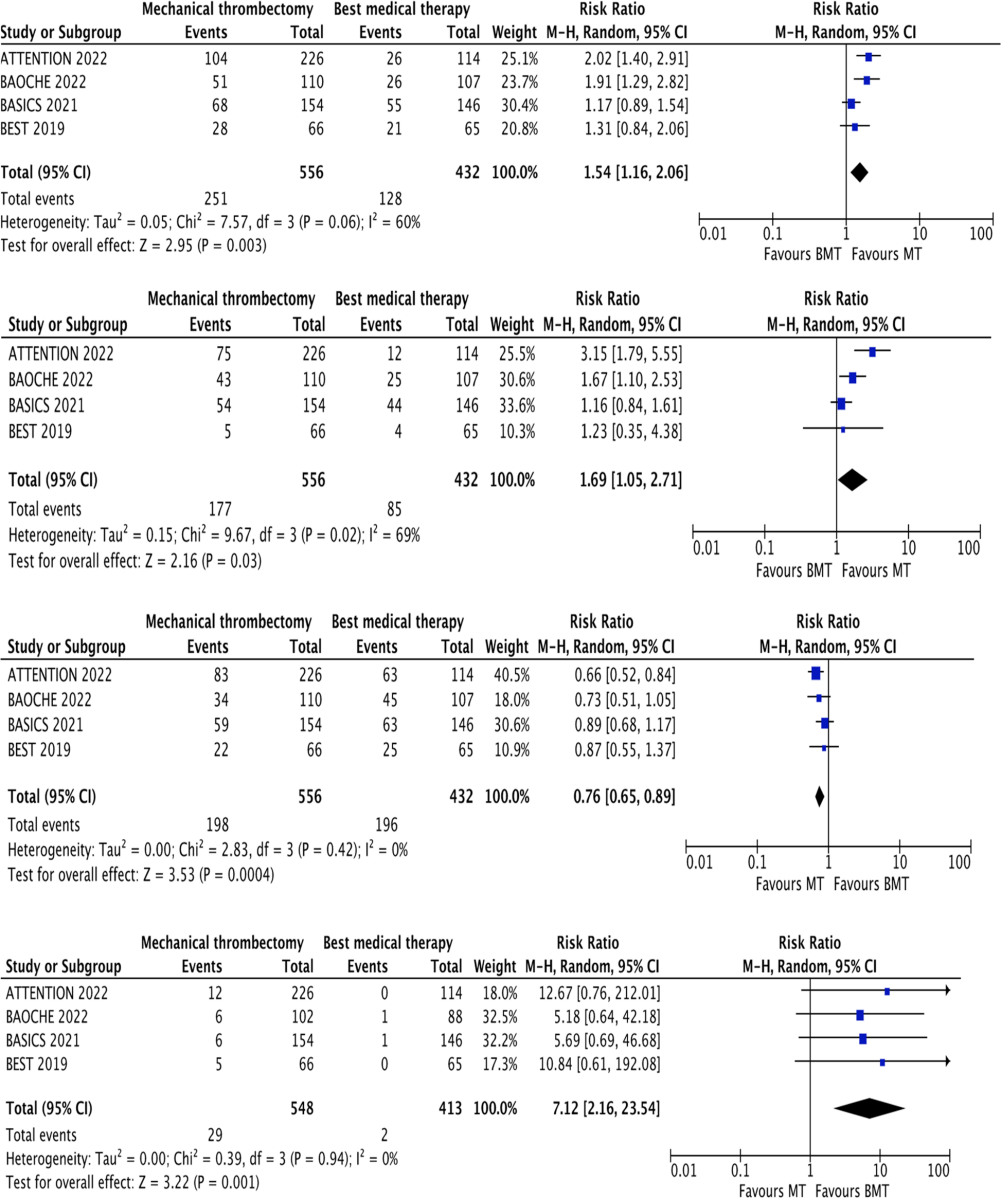
Forest plot of primary and secondary outcomes

We performed a random effects meta-analysis of the four randomized controlled trials (See Fig. 2: Forest plot of primary and secondary outcomes) comprising a total number of 988 participants [5, 6, 8, 10]. 556 were included in the thrombectomy arm verses vs 432 to best medical therapy. We found a statistically significant benefit in the number of patients with a good functional outcome (mRS 0–3) (RR 1.54, 1.16–2.06, *p* = 0.003) and functional independence (mRS 0–2) (RR 1.69, 1.05–2.71, *p* = 0.03) who were treated with thrombectomy when compared to best medical therapy. Thrombectomy was associated with a higher level of sICH (RR 7.12, 2.16–23.54, *p* = 0.001) but this was not reflected in the mortality rate, in contrast MT was associated with a lower mortality rate (RR 0.76, 0.65–0.89, *p* = 0.0004) confirming the benefit of thrombectomy in BAO.

### Risk of bias assessment

**Fig. 3:**
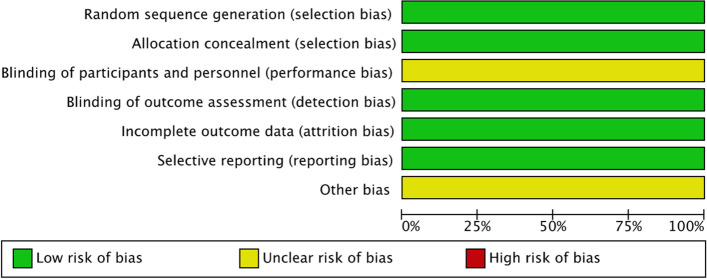
Risk of bias graph

We performed a risk of bias assessment as per the criteria outlined in the Cochrane Handbook for Systematic Reviews of Interventions [16]. A risk of bias graph is presented below (Fig. 3). We did not exclude any studies based on the level of bias found. All four of the included studies were judged at low risk of selection and allocation bias, as they were randomized controlled trials with adequate allocation concealment. Outcome assessment was blind in all four studies and there was minimal loss to follow up. We did not identify any reporting bias. Given the invasive nature of the intervention there was no blinding of the participant or personnel to the intervention, however given the objective nature of the outcomes we deemed this to be at a low risk of bias.

We do note that two studies BEST [5] and BASICS [6] suffered with poor recruitment and high crossover rates which may have introduced bias and diluted the treatment effect. Two of the studies have not been formally published and therefore we have deemed these to be at ‘unclear risk’ until final review of the published data. Overall, we deemed the included studies to be high quality randomized controlled trials with minimal bias.

## Discussion

There is now evidence to support the use of thrombectomy in patients with an acute basilar occlusion with improved disability rates and mortality despite higher rates of sICH. We deemed the trials of good quality with randomization and blinding to outcome assessment appropriately performed.

We found no statistical heterogeneity in the sICH and mortality data. We found moderate statistical heterogeneity in the disability outcome measures but not on visual inspection, we therefore deemed the trials similar enough to combine in a random effects meta-analysis.

Due to the number of trials being too small and lacking a varied sample size for a meaningful analysis, the included studies did not fit the criteria for funnel plot analysis proposed by the Cochrane Collaboration Handbook [16]. We ensured that publication bias was minimized by reviewing conference abstracts and clinical trial registers in order to identify trials conducted but unpublished.

There were large numbers of screened patients treated outside of the trial protocol in BASICS (29%) and BEST (55%) eluding to a degree of bias in the study population and high crossover rates in the BEST trial (22% in the medical group) which may have diluted the size of the treatment effect in these trials [5, 6], however, the ATTENTION and BAOCHE trials had rapid recruitment with minimal crossover rates [8, 10].

Certain questions remain unanswered such as the role of thrombolysis and optimal time windows in order to achieve reperfusion. Two of the included trials enrolled late presenting patients (8–24 hours) [5, 10] and there was variability in the number of patients thrombolysed with BASICs thrombolysing up to 78% of enrolled patients compared to 15–23% in the BAOCHE [6, 10].

We identified a higher rate of patients with symptomatic intracranial hemorrhage in the intervention group in all of the included studies. The overall rates of sICH were low in both groups (4–8% in the intervention group and 0–1% in the medically treated group) and similar to that seen in the anterior circulation [2]. Surprisingly, the increase in the rates of sICH was not reflected in an overall increase in mortality, conversely a lower mortality rate was found in the intervention group reflecting the overall benefit of mechanical thrombectomy in basilar artery occlusions and the high mortality rate in untreated occlusions.

Our study is limited by several factors, firstly two of the studies [8, 10] included are from unpublished, presented data which may lack the detail found from reviewing the full results of the trials. Secondly, we found a moderate level of heterogeneity in the analysis of the mRS outcomes. This may reflect clinical heterogeneity given the different time windows used which may also reflect the variation in thrombolysis rates.

Thirdly, three of the studies were conducted in China which is known to have a higher degree of patients with intracranial atherosclerotic disease [17] (BEST (52%) and BASICS (33%)) which we can speculate may affect the generalizability of the results in a Western population [5, 6, 10]. We suggest an individual patient data analysis is performed once the full trial results are available, to better understand the role of timings, prognostic characteristics and thrombolysis in BAO.

## Conclusion

The long-awaited question of whether to use MT in BAO has now been answered.

MT is associated with lower rates of disability and death in BAO despite an increase risk of sICH. We await the full publication of the ATTENTION and BAOCHE, however given the critical nature of basilar artery occlusion we put forward the results for dissemination.

## Supplementary Information

**Additional file 1.**

## Data Availability

The datasets used and/or analyzed during the current study are available from the corresponding author on reasonable request.

## Abbreviations

BAO: Basilar Artery Occlusion
BMT: Best medical therapy
MT: Mechanical thrombectomy
mRS: Modified Rankin Scale
NIHSS: National Institute of Health Stroke Scale
CTA: Computed tomography angiography
MRA: Magnetic resonance angiography
TICI: Thrombolysis in Cerebral Infarction
PRISMA: Preferred Reporting Items for Systematic Reviews and Meta-Analyses
RR: Relative Risk or Risk Ratio
CI: Confidence Interval
ICH: Intracranial Hemorrhage
sICH: Symptomatic Intracranial Hemorrhage
