# Correction: Mechanical thrombectomy in acute basilar artery stroke: a systematic review and Meta-analysis of randomized controlled trials

**DOI:** 10.1186/s12883-022-03015-3

**Published:** 2022-12-20

**Authors:** Abid Malik, Brian Drumm, Lucio D’Anna, Isabelle Brooks, Benjamin Low, Oishik Raha, Khawar Shabbir, Orsolya Vittay, Joseph Kwan, Zoe Brown, Omid Halse, Sohaa Jamil, Dheeraj Kalladka, Marius Venter, Harri Jenkins, Neil Rane, Abhinav Singh, Maneesh Patel, Charles Hall, Gavin Fatania, Dylan Roi, Kyriakos Lobotesis, Soma Banerjee

**Affiliations:** Stroke Centre, Department of Stroke and Neuroscience, Charing Cross Hospital, Imperial College London NHS Healthcare Trust, Fulham Palace Road, London, W6 8RF UK

**Correction: BMC Neurol 22, 415 (2022)**

**https://doi.org/10.1186/s12883-022-02953-2**

Following publication of the original article [1], the authors reported an error in the Fig. 2. The titles for each of the graphs have been removed. The correct Fig. 2 is shown below.

**Fig. 2:**
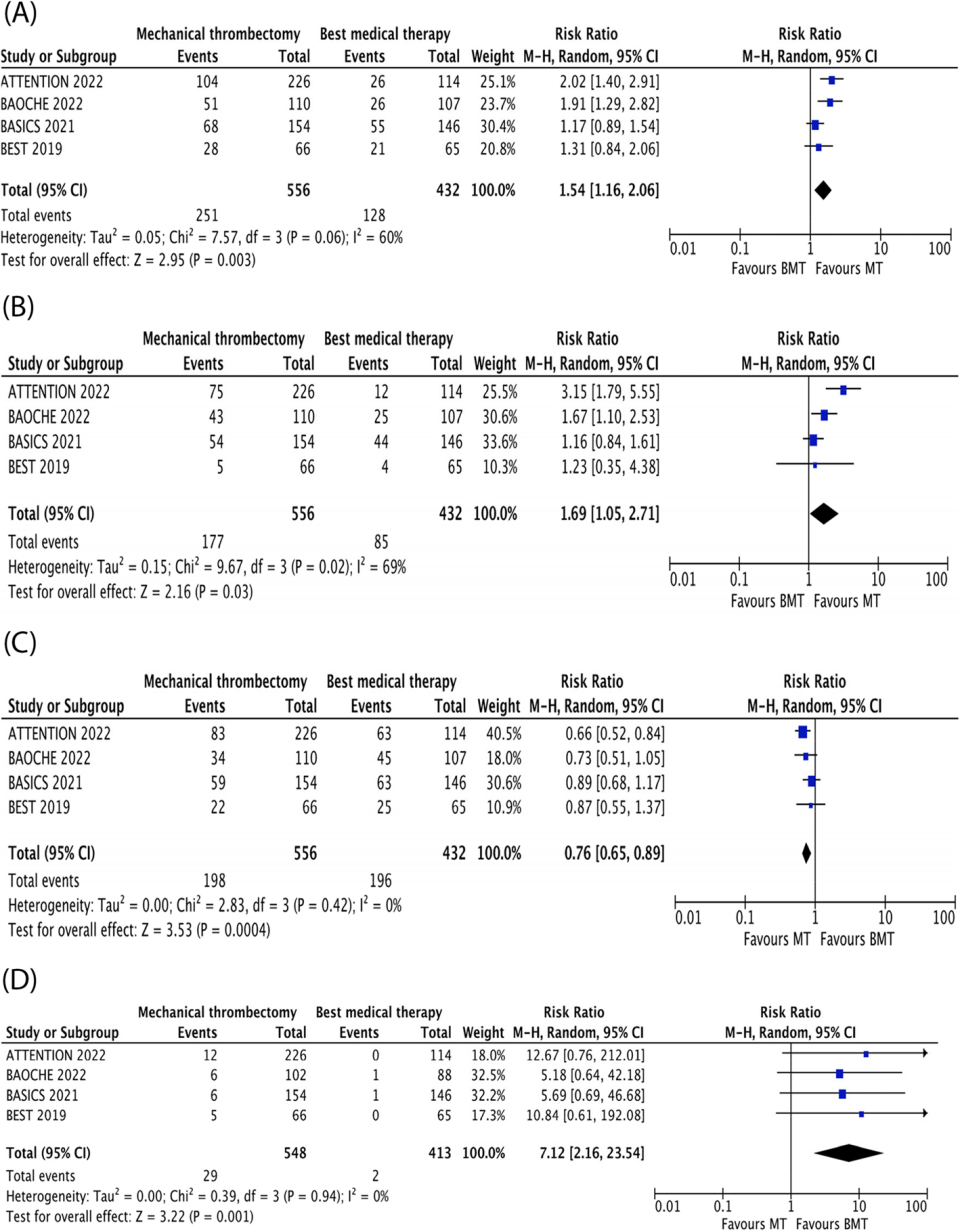
Forest plots of primary and secondary outcomes. **A** Good functional outcome (mRS 0-3) **B** Functional independence (mRS 0-2) **C** Mortality **D** sICH

The original article [1] has been updated.

## References

[1] Malik A, Drumm B, D’Anna L (2022). Mechanical thrombectomy in acute basilar artery stroke: a systematic review and Meta-analysis of randomized controlled trials. BMC Neurol.

