# Supplementary material for: Mechanical thrombectomy in acute basilar artery stroke: a systematic review and Meta-analysis of randomized controlled trials
Source: BMC Neurol. 2022 Nov 9;22:415. doi: 10.1186/s12883-022-02953-2 (PMC9644544; doi:10.1186/s12883-022-02953-2)
Supplement: Supplementary file 1 — Additional file 1. [file 12883_2022_2953_MOESM1_ESM.docx]

**SUPPLEMENTARY MATERIAL**

**Appendix A**

**Search Strategy Pubmed (Central)**

((((((endovascular) OR thrombectomy) AND vertebral) AND posterior circulation) AND vertebrobasilar) AND basilar) AND stroke

(((((endovascular[All Fields] OR ("thrombectomy"[MeSH Terms] OR "thrombectomy"[All Fields])) AND ("spine"[MeSH Terms] OR "spine"[All Fields] OR "vertebral"[All Fields])) AND (posterior[All Fields] AND ("blood circulation"[MeSH Terms] OR ("blood"[All Fields] AND "circulation"[All Fields]) OR "blood circulation"[All Fields] OR "circulation"[All Fields]))) AND vertebrobasilar[All Fields]) AND basilar[All Fields]) AND ("stroke"[MeSH Terms] OR "stroke"[All Fields])
